# Gradual Changes of the Protective Effect of Phenols in Virgin Olive Oils Subjected to Storage and Controlled Stress by Mesh Cell Incubation

**DOI:** 10.1021/acs.jafc.3c04169

**Published:** 2023-10-11

**Authors:** Ana Lobo-Prieto, Noelia Tena, Ramón Aparicio-Ruiz, María Teresa Morales, Diego Luis García-González

**Affiliations:** † Departamento de Química Analítica, Facultad de Farmacia, Universidad de Sevilla, Prof. García González, 2, 41012 Sevilla, Spain; ‡ Pablo de Olavide University, Ctra. de Utrera, km 1, 41013 Sevilla, Spain; § Instituto de la Grasa (CSIC), Edificio 46, Ctra. de Utrera, km 1, 41013 Sevilla, Spain

**Keywords:** antioxidant activity, phenols, pigments, FTIR spectroscopy, virgin olive oil, storage, hydroperoxides

## Abstract

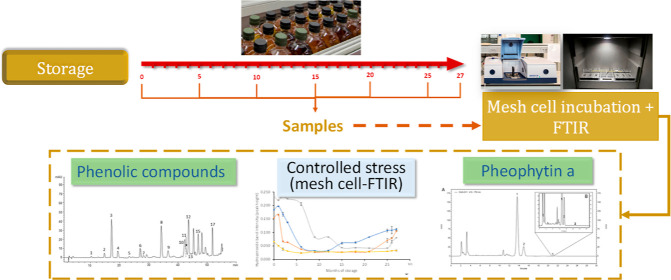

The oxidation reactions that take place in virgin olive oil under moderate conditions involved the combined effect of antioxidant and prooxidant compounds. Given the complexity of oxidation processes of multicomponent matrices, there is still a need to develop new methods with a dynamic approach to study the persistence of the compounds with healthy properties. This work studied the joint evolution of them, including phenols and pheophytin *a*, modeling their tendency during a real storage. The regression equations performed with the total phenol concentration showed that around 2% of the concentration was lost every month. Simultaneously, the progress of oxidation was evaluated by mesh cell incubation and Fourier transform infrared analysis. This method pointed out that, in the presence of light, the prooxidant effect of pigments was able to mask the protective effect of phenols, until the pheophytin *a* concentration was lower than 1 mg/kg. The antioxidant effect of phenols was less remarkable when the concentration loss was 35% or more.

## 1 Introduction

The traditional Mediterranean dietary pattern is considered one of the healthiest and most palatable and sustainable food models.^1^ For that reason, this dietary model is considerably growing, even being recognized as an intangible cultural heritage of humanity by UNESCO in 2010.^2,3^ Among other foods, virgin olive oil (VOO) is an important ingredient of this diet because its daily intake is related to the prevention of cardiovascular and other chronic diseases, such as diabetes.^4^ These characteristics, together with the proven benefits of the Mediterranean diet,^5^ have made an increase in consumption worldwide in the past decade and the international transactions, such as extra-EU exports, are also rising.^6^ As it is well reported in the literature, the nutritional and healthy properties of VOO are assigned to its antioxidant compounds, mainly phenolic compounds and tocopherols,^4,7^ among others. In fact, olive oil phenols have the health claim granted by the European Union, which ensures that a quantity higher than 250 mg/kg of these compounds present in the oil has a beneficial effect on human health.^8^ The demonstrated healthy effect^9^ and the sensory contributions of phenolic compounds^10,11^ to VOO make this food product highly demanded and appreciated by consumers. This aspect, together with the fact that consumers are increasingly concerned about the nutritional characteristics of the foods that they consume on a daily basis, makes it necessary to guarantee the persistence of these compounds during the shelf life of the oil for both producers and consumers.

The interest of the preservation of the antioxidant compounds, phenols and tocopherols, in the VOO is also referred to the fact that they are closely linked to the VOO stability and its resistance to oxidation.^12^ Previous studies^13,14^ have suggested that the stability of VOOs is highly correlated with phenols. Since phenols act as chain-break antioxidants^11,15^ and they inhibit the decomposition reactions by different pathways,^16,17^ they provide the oil a high resistance to oxidation compared to other edible oils. On the other hand, it contains other minor components with pro-oxidant effects in the presence of light, such as chlorophyll pigments. They can modify the oxidative stability of oils,^18,19^ promoting the oxidation through the generation of singlet oxygen in the presence of light or acting as an antioxidant in the dark.^20,21^ Along the distribution, storage, and commercialization period, VOO is inevitably subjected to the oxidation process, which could modify the initial quality of the VOOs and also reduce their nutritional and healthy quality.^22^ Even under moderate and controlled storage conditions, the oil may undergo small changes in the concentration of these minor compounds, antioxidants, and prooxidants, which modifies the initial stability of the oil and even the best before date estimated by producers.

The quality parameters established in the trade standards report on the degradation state of the oil expressed as a concentration or value of a physical–chemical parameter,^23^ such as peroxide value (PV) or spectroscopy absorbance.^24^ The degradation of the oils can also alter the concentration of some antioxidant compounds, such as phenols. Although the standard methods are simple and adaptable for routine analysis, they just give information at the time of the measurement without providing any clue about their tendency during the shelf life of the product and their impact on the oil stability. Furthermore, they are costly, time-consuming, and nonenvironmentally friendly;^25^ consequently, they are difficult to implement for monitoring the changes of VOO quality during the commercialization period. In order to ensure that the VOOs reach consumers with the expected quality and healthy properties, the development of new methods with a dynamic approach is still needed. The high number of reactions involved in the oxidation process, even under moderate conditions of light and temperature, and the complexity of the mixtures of antioxidant and prooxidant compounds that form the VOO matrix make difficult the prediction of the kinetics or evolution of the oil quality and the concentration of the antioxidant compounds to comply with the health claim indicated in the label.

For that reason, spectroscopic techniques are pointed out as a promising alternative,^26–32^ rapid and green analysis, to carry out in situ studies where a global and dynamic perspective of the VOO degradation state can be acquired. Previous studies^27,33^ verified the applicability of the Fourier transform infrared (FTIR) accessory called mesh cell to track the chemical degradation of VOO through the monitoring of the intensity of the band assigned to different oxidation products,^27,34^ which are interpreted as oxidation markers. A mesh cell is able to accelerate the oxidation process by subjecting the oil to a controlled stress by incubation under similar conditions to those found in supermarkets.^27^ This method allows tracking the oxidation state of the oils during their shelf life, providing rapid and representative information about the chemical response of the oils to moderate conditions.^35^

In this context, the aim of this work was to study the joint evolution of antioxidant and pro-oxidant compounds present in VOO during its degradation by means of a storage experiment designed to emulate the normal conditions found in a supermarket. Particularly, the changes of the individual phenols, as main antioxidants, and pheophytin *a*, as a pro-oxidant when light is involved, were studied in monovarietal VOOs with the aim of modeling their trends and evaluating their linear responses during the storage. One of the questions that arises when studying the changes in antioxidant and pro-oxidant compounds over time is how to evaluate the actual effect of those changes on the oil stability over time. For that reason, the chemical information was complemented with an FTIR spectroscopy study of the samples in which the oils were subjected to moderate conditions of heating and light and the spectral changes were evaluated. Thus, the resistance of VOOs to oxidation under moderate conditions was assessed by an accelerated method based on mesh cell incubation and FTIR analysis. VOOs with different concentration ranges of phenols and pigments were subjected to storage and controlled stress by mesh cell incubation under different moderate conditions. During incubation, the spectral band assigned to primary oxidation products (hydroperoxides) was monitored and used as a spectroscopic marker to inform on the oxidation resistance. The relation between the spectral changes registered and the time-trend changes of pigments and phenolic compounds, especially those that computed to the health claim established by the EU regulation, was evaluated through multivariate analysis.

## 2 Materials and Methods

### 2.1 Samples and Storage Conditions

The selection of the VOO samples was focused on covering the wide range of phenol content in VOOs, samples with high (≥500 mg/kg), intermediate (499–300 mg/kg), and low concentration (299–200 mg/kg).^10^ For that reason, the four monovarietal VOOs were collected from three different olive cultivars (Picual, Hojiblanca, and Arbequina), which were directly provided by Andalusian producers at the time of production. In fact, the samples were collected at the olive oil mills just at the moment of the production. This moment was considered to be time zero and the maximum freshness level. The codes used to identify the VOOs and their respective cultivars were as follows: VOO1, Hojiblanca; VOO2, Arbequina-1; VOO3, Picual; and VOO4, Arbequina-2. The samples were transported under controlled conditions to the laboratory. Temperature was lower than 20 °C during transport, and the oils were always protected from light. Then, once the samples were in the laboratory, each oil was transferred to 28 bottles of 500 mL of transparent poly(ethylene terephthalate) provided by producers. The storage experiment of the four VOOs was performed by using one bottle for the fresh oil (the “time zero”) and one bottle for each month of storage (27 months). During the 27 months of storage, the samples were exposed to room temperature (23 ± 7 °C) and a light intensity of 1000 lx in 12 h light/dark cycles, simulating the conditions of a supermarket shelf under controlled conditions of temperature and humidity. The maximum and minimum values of temperatures were 29.7–16.3 °C and humidity were 70–21%. At the end of each month, a new bottle per oil subjected to storage was analyzed and discarded afterward.

The fresh samples were categorized according to the European Commission regulation,^36^ prior to the storage experiment. Three of the four VOOs studied (VOO1, VOO2, and VOO3) belonged to the “extra VOO” category, whereas VOO4 was categorized as “VOO” because panelists detected a winey-vinegary defect (median of defect = 2.1). According to the results obtained for free fatty acids (FFAs), PV, and UV absorbance (K270 and K232), VOO2 was the most oxidized oil when bottling, showing the highest values of these parameters. These data are available in an already published data set.^37^ Furthermore, during the storage period [27 months at room temperature (23 ± 7 °C) and a light intensity of 1000 lx in 12 h light/dark cycles], the quality parameters were determined in the stored samples every month following the methods for chemical analysis of olive oil, which are specified in “Trade standard applying to olive oils and olive pomace oils (COI/T.15/NC no. 3/Rev. 19 2022)”.^23^

### 2.2 Mesh Cell Incubation and FTIR Analysis

**Figure 1:**
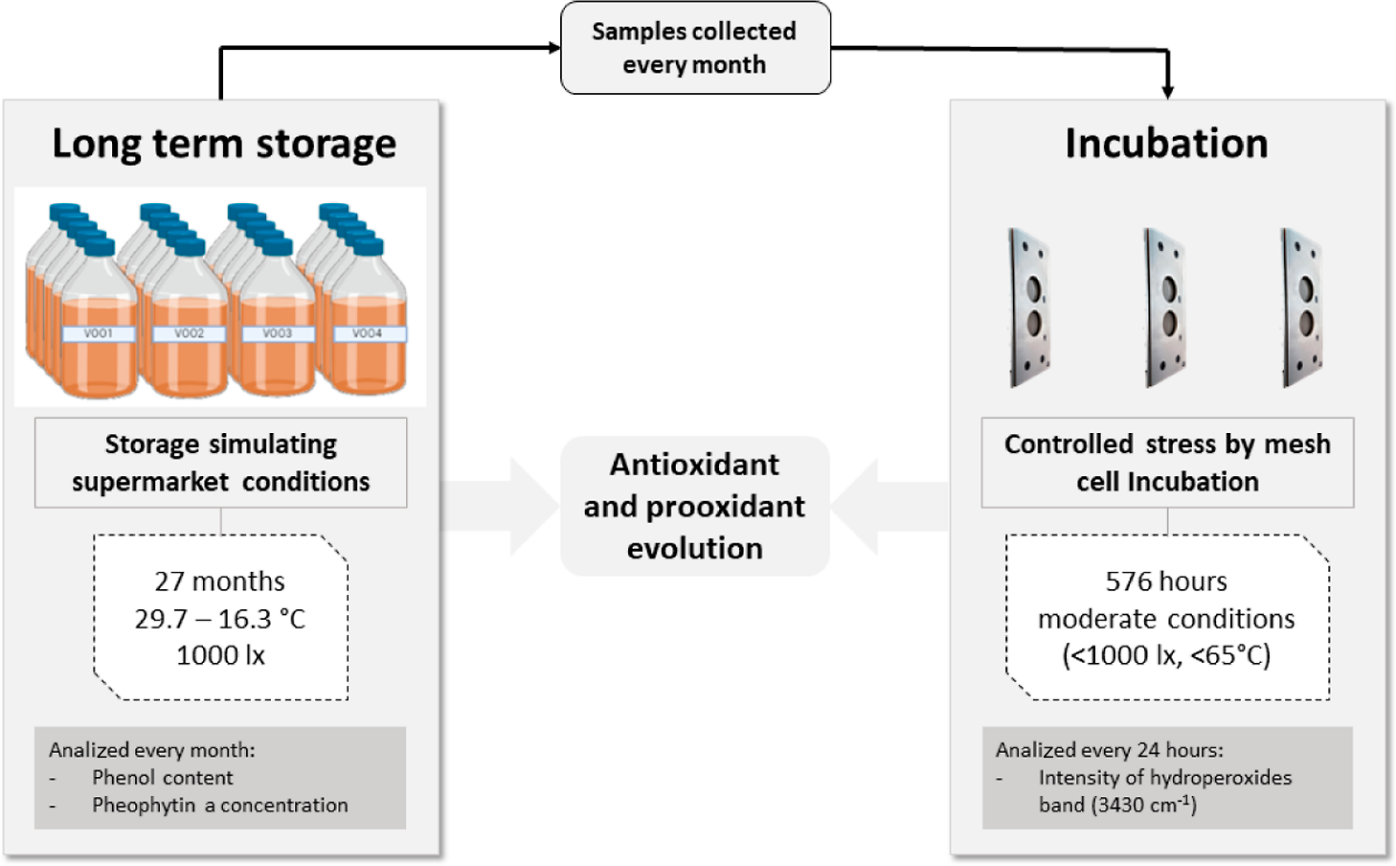
Scheme of the experimental design.

The oil sample (16 μL) was loaded and uniformly distributed on the mesh in one of the two apertures of the cell using a micropipette. The loaded mesh cells were incubated in a compartment designed for that,^38^ where the cells were horizontally exposed for 576 h to a controlled stress under three different moderate conditions: (1) in the dark and at 35 °C, (2) at 400 lx and 23 °C, and (3) 400 lx and 35 °C. These conditions were previously selected.^19,27,33^ During 576 h of incubation, the spectral changes of the band assigned to hydroperoxides, located at 3430 cm^–1^,^27^ were monitored by FTIR spectroscopy every 24 h, and the spectra before incubation (0 h) were also acquired. The experiments were performed in triplicate. The controlled stress by mesh cell incubation was carried out with the fresh samples and also with the samples collected during storage (13 samples per VOO). Figure 1 shows a scheme of the experimental design applied.

The FTIR analyses were carried out with a Bruker Vertex 70 FTIR spectrometer (Bruker, Optics, Germany) equipped with a deuterated triglycine sulfate (DTGS) detector. The mesh cell accessory was designed to be inserted in the transmission cell holder provided with the instrument, thus allowing spectrum acquisition. All the spectra were collected in the range of 5000–400 cm^–1^ by coaddition of 32 scans with a resolution of 4 cm^–1^ and using weak Norton–Beer apodization. OPUS software version 7.2 (Bruker Optics, Ettlingen, Germany) was used for collecting and manipulating the spectra, which were normalized to an effective path length of 110 μm to allow for the comparison between samples.

The FTIR spectra were normalized according to the procedure described by Tena et al.^27^ The intensity of the hydroperoxide band was expressed as a peak height value, which was measured relative to a selected single-point baseline at 3324 cm^–1^ by implementing macros programmed with the Macro/Basic tool provided by Omnic 7.3 (Thermo Electron Inc., Madison, WI, USA).

### 2.3 Determination of Phenolic Compounds

The phenolic composition was determined following the method described by Aparicio-Ruiz et al.^39^ The oil sample (2.5 g) was diluted in 6 mL of hexane together with the internal standards *p*-hydroxyphenylacetic (0.12 mg/mL) and *o*-coumaric (0.01 mg/mL) (Merck KGaA, Darmstadt, Germany). The isolation of the phenolic fraction was carried out with methanol by solid-phase extraction using diol-bonded phase cartridges. The high-performance liquid chromatography (HPLC) system (Agilent Technologies 1200, Waghaeusel–Wiesental, Germany) was equipped with a diode array detector and a LiChrospher 100RP-18 column (4.0 mm i.d. × 250 mm; 5 μm, particle size) (Merck KGaA, Darmstadt, Germany). The solvent used was a mixture of water/*ortho*-phosphoric acid (99.5:0.5 v/v) (solvent A) and methanol/acetonitrile (50:50 v/v) (solvent B), with a flow rate of 1.0 mL/min. The gradient elution of the solvent was programed as from 95% (A)–5% (B) to 70% (A)–30% (B) in 25 min; 65% (A)–35% (B) in 10 min; 60% (A)–40% (B) in 5 min; 30% (A)–70%(B) in 10 min and 100% (B) in 5 min, followed by 5 min of maintenance. The chromatographic signals were obtained at 235, 280, and 335 nm. The quantification of the phenols, cinnamic acid, and lignans was carried out at 235 and 280 nm using *p*-hydroxyphenylacetic acid as the internal standard. The quantification of flavones was done at 335 nm by using *o*-coumaric acid as the internal standard. Figure S1 shows the phenolic compounds identified in the HPLC chromatograms. The response factors and recoveries were based on the procedure developed by Mateos et al.^40^ The analyses were carried out in duplicate.

### 2.4 Determination of Pheophytin *a*

The determination of the degradation products of chlorophyll *a*, such as pheophytin *a*, was based on the standard method ISO 29841:2016.^41^ A LaChrom Elite HPLC system (Hitachi, Tokyo, Japan) equipped with a diode array detector and a Lichrospher RP18 HPLC column, 250 mm length, 4.0 mm internal diameter, filled with reversed-phase, particles size 5 μm (Merck, Darmstadt, Germany), was used to carry out the analysis. The quantification was carried out by a calibration curve. Thus, seven different volumes of the standard solution, which varied from 1 to 75 μL, were injected into the HPLC system. The calibration factors of pheophytin *a* were calculated from the slope values of their calibration curves. The expression of the results was expressed in concentration values (mg/kg). As an example, Figure S2 (Supporting Information) shows a chromatogram obtained in the pigment analysis. The analyses were performed in duplicate.

### 2.5 Data Processing and Multivariate Analysis

The STATISTICA 8 package (Statsoft, Tulsa, OK, USA) was used to carry out statistical analysis. In the case of phenols, analysis of variance (ANOVA) was applied to identify significant differences between the concentration means computed in different storage periods. Microsoft Excel 2019 Statistical Software was used to perform the linear regression of some phenols with respect to the storage months.

Principal component analysis (PCA) was carried out with the concentration values of individual phenolic compounds and pheophytin *a* during the storage of the four oils to explore the data from a multivariate perspective. Furthermore, the intensity values of the hydroperoxide band during 27 months of storage and after 576 h of controlled stress under the different moderate conditions were included in the projection as additional variables to support the relationship between the analyzed compounds and the observed changes in this band. The multivariate analysis was performed with the mean spectra obtained from triplicate analyses.

A partial least-square (PLS) model was performed with the FTIR spectra and the concentration of the phenolic compounds that computed to the health claim (hydroxytyrosol, tyrosol, hydroxytyrosol acetate, tyrosol acetate, 3,4-DHPEA-EDA, *p*-HPEA-EDA, 3,4-DHPEA-EA, and *p*-HPEA-EA) per each VOO stored. The calibration models were built by using the spectra obtained during VOO storage under moderate conditions and after 576 h of controlled stress in mesh cell incubations carried out with light: 400 lx and 23 °C and 400 lx and 35 °C. Each PLS calibration model was developed using a training set and a test set. The training set, which was used for developing the calibration models, contained 13 spectra obtained during the storage and after 576 h of incubation in the mesh cell. The test set was used for testing the prediction ability of the models, and it contained seven spectra randomly selected from the storage of the same VOOs and that were not used for developing the calibration models. The performance of the regression models was evaluated by means of an internal validation (cross-validation, CV) and an external validation (independent test samples, P) using the coefficients of determination (*R*_CV_^2^ and *R*_P_^2^). The *R*_CV_^2^ value defined the ability of the spectra to represent the changes occurred in the concentration of the phenolic compounds that computed to the health claim during storage, while the *R*_P_^2^ value defined the ability of the model to predict them. The latter value allowed testing of the robustness of the model. The error of a calibration was defined as the root-mean-square error for cross-validation (RMSECV) and the prediction error (RMSEP) when external validation was used. The optimal number of latent variables was selected as that minimizes the root-mean-square errors (RMSECV and RMSEP). In addition, the relative predictive deviation (RPD_CV_ and RPD_P_) was used to compare the accuracy of the models. This coefficient was interpreted according to Nicolaï et al.^42^

## 3 Results and Discussion

### 3.1 Study of Phenols and Pheophytin *a* in Fresh and Stored VOOs

Table 1 shows the concentration of the individual phenols identified and quantified in the stored oils on a quarterly basis for 27 months of storage. Furthermore, the total concentrations of phenols per sample are also showed. According to the total concentration of phenols in the fresh oils (storage month 0 in Table 1), VOO3 showed the highest concentration (534.82 mg/kg), followed by VOO4 (451.25 mg/kg), VOO2 (338.90 mg/kg), and VOO1 (226.71 mg/kg) (Table 1). As it was expected,^43,44^ the most abundant phenolic compounds were secoiridoid derivatives, which corresponded to 79, 90, 96, and 95% of the total concentration of phenols for VOO1, VOO2, VOO3, and VOO4, respectively. Furthermore, the fresh oils showed higher concentration of the dialdehydic forms of elenolic acid (3,4-DHPEA-EDA, *p*-HPEA-EDA) than the aldehydic forms (3,4-DHPEA-EA, *p*-HPEA-EA), except for VOO3 which showed the inverse situation (Table 1). On the other hand, VOO2 showed the highest initial concentration of simple phenols, which was at least 3 times higher than the concentration quantified in the other three oils (Table 1).

**Table 1:** Concentration of the Individual Phenols (Milligrams per Kilogram, Mean ± Standard Deviation) Identified and Quantified in Each Stored Oil on a Quarterly Basis during 27 Months of Storage^a^

phenolic compounds	storage months									
	0	3	6	9	12	15	18	21	24	27
**VOO1**										
hydroxytyrosol	0.49 ± 0.01	0.82 ± 0.02	1.07 ± 0.02	1.33 ± 0.01	1.64 ± 0.02	1.70 ± 0.06	2.02 ± 0.02	2.20 ± 0.03	2.19 ± 0.01	2.27 ± 0.02
tyrosol	2.20 ± 0.01	2.50 ± 0.12	3.02 ± 0.09	3.40 ± 0.04	3.61 ± 0.06	3.77 ± 0.11	4.16 ± 0.05	4.48 ± 0.03	4.24 ± 0.19	4.55 ± 0.04
vanillic acid	0.88 ± 0.02	0.99 ± 0.01	0.99 ± 0.02	0.93 ± 0.02	0.95 ± 0.02	0.92 ± 0.02	0.98 ± 0.01	0.98 ± 0.06	0.85 ± 0.01	0.87 ± 0.02
vanillin	0.15 ± 0.01	0.15 ± 0.01	0.14 ± 0.01	0.14 ± 0.01	0.13 ± 0.01	0.13 ± 0.01	0.13 ± 0.01	0.12 ± 0.01	0.11 ± 0.01	0.12 ± 0.01
*p*-coumaric acid	0.90 ± 0.03	0.86 ± 0.04	0.82 ± 0.02	0.82 ± 0.03	0.83 ± 0.01	0.82 ± 0.01	0.81 ± 0.01	0.78 ± 0.01	0.69 ± 0.04	0.58 ± 0.02
hydroxytyrosol acetate	8.17 ± 0.14	7.09 ± 0.15	6.03 ± 0.14	4.97 ± 0.10	3.82 ± 0.12	2.90 ± 0.29	1.97 ± 0.15	1.66 ± 0.17	1.23 ± 0.14	1.01 ± 0.15
3,4-DHPEA-EDA	28.01 ± 1.54	26.66 ± 1.45	23.03 ± 1.50	19.55 ± 2.24	14.08 ± 2.19	10.03 ± 0.76	7.55 ± 1.32	5.04 ± 2.20	5.22 ± 1.32	4.38 ± 1.45
*p*-HPEA-EDA	75.07 ± 1.48	83.11 ± 2.25	70.08 ± 1.48	61.75 ± 2.19	48.09 ± 2.20	39.55 ± 2.28	35.02 ± 2.05	30.81 ± 0.57	28.47 ± 1.30	26.96 ± 1.44
pinoresinol	1.34 ± 0.10	1.56 ± 0.04	1.64 ± 0.11	1.76 ± 0.23	1.91 ± 0.03	2.13 ± 0.16	2.41 ± 0.09	2.59 ± 0.10	2.66 ± 0.05	2.62 ± 0.12
cinnamic acid	1.34 ± 0.02	1.69 ± 0.10	1.73 ± 0.10	1.58 ± 0.08	1.47 ± 0.09	1.43 ± 0.14	1.55 ± 0.10	1.58 ± 0.11	1.42 ± 0.04	1.31 ± 0.11
acetoxypinoresinol	1.53 ± 0.05	1.09 ± 0.09	1.02 ± 0.09	0.93 ± 0.12	0.91 ± 0.06	0.90 ± 0.07	0.88 ± 0.07	0.86 ± 0.06	0.85 ± 0.06	0.75 ± 0.13
3,4-DHPEA-EA	53.49 ± 2.21	53.02 ± 1.48	46.25 ± 2.07	41.93 ± 2.76	33.61 ± 4.33	30.82 ± 1.40	24.73 ± 2.07	22.52 ± 2.20	21.99 ± 1.59	19.70 ± 1.34
*p*-HPEA-EA	22.55 ± 1.33	25.69 ± 2.03	25.07 ± 1.46	22.78 ± 2.09	22.04 ± 1.49	23.07 ± 1.44	24.53 ± 0.83	23.16 ± 1.45	22.27 ± 1.53	19.30 ± 1.49
luteolin	18.90 ± 1.36	15.77 ± 0.73	14.22 ± 1.48	12.36 ± 1.33	9.19 ± 1.39	6.96 ± 1.33	5.40 ± 0.83	4.31 ± 0.58	3.71 ± 0.77	3.79 ± 0.76
apigenin	10.42 ± 0.64	8.79 ± 1.29	8.06 ± 1.47	7.18 ± 0.84	5.15 ± 0.78	4.00 ± 0.77	3.31 ± 0.81	3.12 ± 0.90	2.24 ± 0.43	2.29 ± 0.18
total concentration	225.44 ± 8.48	229.81 ± 9.51	203.19 ± 6.60	181.41 ± 6.89	147.43 ± 12.59	129.11 ± 3.73	116.25 ± 4.22	104.31 ± 7.82	97.84 ± 7.33	90.50 ± 6.33
**VOO2**										
hydroxytyrosol	12.95 ± 0.62	13.12 ± 0.59	13.73 ± 0.21	13.96 ± 0.41	14.09 ± 0.28	14.42 ± 0.15	14.82 ± 0.18	15.19 ± 0.12	15.40 ± 0.16	15.71 ± 0.19
tyrosol	3.83 ± 0.12	4.08 ± 0.29	4.55 ± 0.23	4.72 ± 0.18	4.77 ± 0.18	4.85 ± 0.21	4.98 ± 0.22	5.16 ± 0.27	5.19 ± 0.19	5.20 ± 0.38
vanillic acid	0.21 ± 0.01	0.21 ± 0.01	0.22 ± 0.01	0.20 ± 0.01	0.20 ± 0.01	0.17 ± 0.01	0.20 ± 0.01	0.20 ± 0.01	0.20 ± 0.01	0.20 ± 0.01
vanillin	0.27 ± 0.01	0.25 ± 0.03	0.24 ± 0.01	0.24 ± 0.01	0.23 ± 0.01	0.21 ± 0.01	0.21 ± 0.01	0.20 ± 0.01	0.19 ± 0.01	0.18 ± 0.01
*p*-coumaric acid	0.24 ± 0.02	0.22 ± 0.01	0.21 ± 0.01	0.21 ± 0.01	0.21 ± 0.01	0.21 ± 0.02	0.21 ± 0.01	0.22 ± 0.01	0,23 ± 0.01	0.23 ± 0.02
hydroxytyrosol acetate	0.35 ± 0.03	0.08 ± 0.02	0.06 ± 0.01	0.06 ± 0.01	0.04 ± 0.01	0.09 ± 0.01	0.07 ± 0.01	0.07 ± 0.01	0.08 ± 0.01	0.10 ± 0.01
3,4-DHPEA-EDA	85.11 ± 2.97	93.07 ± 1.55	84.26 ± 2.27	78.49 ± 1.30	74.46 ± 2.14	68.18 ± 0.63	63.21 ± 1.54	60.99 ± 0.84	60.08 ± 1.33	60.62 ± 0.88
*p*-HPEA-EDA	117.11 ± 2.20	118.04 ± 1.28	106.18 ± 2.30	95.75 ± 1.51	82.14 ± 0.80	70.18 ± 2.25	55.02 ± 0.45	46.56 ± 0.81	42.37 ± 1.33	39.02 ± 0.62
pinoresinol	3.02 ± 0.02	2.82 ± 0.05	2.97 ± 0.10	3.14 ± 0.07	3.29 ± 0.13	3.57 ± 0.04	3.80 ± 0.07	4.02 ± 0.05	4.22 ± 0.04	4.21 ± 0.07
cinnamic acid	2.47 ± 0.12	2.46 ± 0.04	2.38 ± 0.06	2.39 ± 0.21	2.26 ± 0.14	2.33 ± 0.12	2.32 ± 0.04	2.39 ± 0.06	2.31 ± 0.12	2.27 ± 0.15
acetoxypinoresinol	2.38 ± 0.05	2.30 ± 0.09	2.37 ± 0.06	2.61 ± 0.13	2.36 ± 0.09	2.63 ± 0.14	2.48 ± 0.08	2.92 ± 0.06	2.89 ± 0.05	2.83 ± 0.07
3,4-DHPEA-EA	78.40 ± 1.29	67.07 ± 2.18	62.72 ± 1.34	53.06 ± 2.27	45.05 ± 2.19	40.13 ± 0.85	37.67 ± 1.14	32.36 ± 0.53	29.14 ± 1.36	26.66 ± 1.32
*p*-HPEA-EA	22.76 ± 0.19	20.29 ± 0.77	19.62 ± 1.50	17.20 ± 1.32	17.04 ± 0.84	16.23 ± 0.81	17.12 ± 0.13	17.53 ± 0.79	17.98 ± 0.78	17.59 ± 0.79
luteolin	7.72 ± 0.22	7.33 ± 0.17	6.91 ± 0.47	6.61 ± 0.40	6.03 ± 0.21	5.58 ± 0.14	4.96 ± 0.13	4.41 ± 0.23	4.38 ± 0.08	4.50 ± 0.11
apigenin	2.10 ± 0.06	2.11 ± 0.10	1.99 ± 0.09	1.74 ± 0.14	1.63 ± 0.18	1.57 ± 0.09	1.28 ± 0.09	1.11 ± 0.06	1.11 ± 0.07	1.14 ± 0.16
total concentration	338.90 ± 2.99	333.44 ± 5.55	308.40 ± 2.27	280.30 ± 6.05	253.82 ± 6.17	231.23 ± 0.16	209.29 ± 2.53	193.95 ± 2.11	186.61 ± 3.41	183.81 ± 0.48
**VOO3**										
hydroxytyrosol	2.27 ± 0.17	3.29 ± 0.11	3.63 ± 0.14	4.49 ± 0.30	5.03 ± 0.14	5.72 ± 0.18	6.23 ± 0.08	6.68 ± 0.09	6.88 ± 0.04	7.43 ± 0.35
tyrosol	3.47 ± 0.29	4.19 ± 0.40	4.51 ± 0.34	5.05 ± 0.42	5.85 ± 0.19	6.09 ± 0.19	6.75 ± 0.47	7.05 ± 0.28	7.46 ± 0.27	7.95 ± 0.42
vanillic acid	0.50 ± 0.01	0.52 ± 0.02	0.47 ± 0.02	0.49 ± 0.02	0.52 ± 0.01	0.49 ± 0.01	0.47 ± 0.01	0.44 ± 0.02	0.46 ± 0.02	0.43 ± 0.01
vanillin	0.17 ± 0.01	0.15 ± 0.01	0.15 ± 0.01	0.15 ± 0.01	0.15 ± 0.01	0.14 ± 0.01	0.14 ± 0.01	0.14 ± 0.01	0.13 ± 0.01	0.12 ± 0.01
*p*-coumaric acid	0.41 ± 0.02	0.42 ± 0.02	0.37 ± 0.01	0.35 ± 0.02	0.39 ± 0.04	0.39 ± 0.02	0.37 ± 0.02	0.38 ± 0.01	0.37 ± 0.02	0.36 ± 0.01
hydroxytyrosol acetate	3.40 ± 0.23	2.61 ± 0.18	2.26 ± 0.27	1.95 ± 0.25	1.60 ± 0.13	1.55 ± 0.24	1.29 ± 0.15	1.07 ± 0.11	0.87 ± 0.08	0.83 ± 0.17
3,4-DHPEA-EDA	81.16 ± 0.82	78.70 ± 1.62	72.88 ± 1.14	60.35 ± 3.63	54.58 ± 2.04	49.60 ± 0.81	40.62 ± 3.44	33.71 ± 1.33	33.12 ± 0.97	33.72 ± 0.85
*p*-HPEA-EDA	140.34 ± 2.92	134.82 ± 2.01	118.52 ± 4.76	101.85 ± 6.69	86.31 ± 5.48	78.34 ± 1.53	67.32 ± 2.47	64.32 ± 2.05	64.21 ± 0.62	55.27 ± 2.19
pinoresinol	3.60 ± 0.13	4.15 ± 0.10	4.18 ± 0.09	4.28 ± 0.10	4.34 ± 0.10	4.37 ± 0.13	4.60 ± 0.27	5.28 ± 0.17	5.76 ± 0.41	5.79 ± 0.35
cinnamic acid	1.27 ± 0.02	1.33 ± 0.07	1.34 ± 0.02	1.31 ± 0.02	1.22 ± 0.07	1.06 ± 0.04	0.92 ± 0.05	0.85 ± 0.02	0.74 ± 0.08	0.76 ± 0.06
acetoxypinoresinol	3.22 ± 0.23	2.71 ± 0.21	2.76 ± 0.11	1.57 ± 0.30	1.70 ± 0.17	1.31 ± 0.34	1.36 ± 0.26	1.47 ± 0.11	2.10 ± 0.12	3.25 ± 0.15
3,4-DHPEA-EA	257.29 ± 2.79	247.04 ± 4.98	226.34 ± 7.47	202.24 ± 12.78	173.84 ± 13.84	148.11 ± 11.96	132.49 ± 6.51	105.59 ± 13.78	109.24 ± 7.14	95.51 ± 6.89
*p*-HPEA-EA	32.01 ± 0.76	32.74 ± 0.54	32.15 ± 0.65	28.85 ± 1.33	26.86 ± 1.10	25.19 ± 0.92	25.39 ± 0.47	23.43 ± 1.48	23.58 ± 0.56	23.78 ± 0.76
luteolin	4.22 ± 0.16	3.74 ± 0.15	3.27 ± 0.15	3.20 ± 0.13	2.84 ± 0.28	2.48 ± 0.34	2.14 ± 0.44	1.54 ± 0.24	1.59 ± 0.09	1.59 ± 0.12
apigenin	1.50 ± 0.13	1.28 ± 0.14	1.23 ± 0.11	1.12 ± 0.08	1.10 ± 0.03	0.90 ± 0.04	0.78 ± 0.04	0.67 ± 0.10	0.78 ± 0.05	0.80 ± 0.10
total concentration	534.82 ± 5.35	517.70 ± 6.09	474.05 ± 14.33	417.27 ± 24.32	366.32 ± 22.82	325.69 ± 15.49	290.86 ± 12.85	252.62 ± 18.62	257.29 ± 8.63	237.61 ± 8.53
**VOO4**										
hydroxytyrosol	1.15 ± 0.06	1.98 ± 0.08	2.59 ± 0.14	2.98 ± 0.20	3.50 ± 0.06	3.95 ± 0.13	4.41 ± 0.15	4.66 ± 0.25	5.41 ± 0.35	5.89 ± 0.10
tyrosol	1.23 ± 0.01	1.51 ± 0.01	1.75 ± 0.01	1.88 ± 0.03	2.01 ± 0.07	2.03 ± 0.24	2.06 ± 0.07	2.09 0.03	2.24 ± 0.06	2.35 ± 0.07
vanillic acid	0.07 ± 0.01	007 ± 0.01	0.07 ± 0.01	0.06 ± 0.01	0.06 ± 0.01	0.06 ± 0.01	0.06 ± 0.01	0.06 ± 0.01	0.05 ± 0.01	0.05 ± 0.01
vanillin	0.26 ± 0.01	0.23 ± 0.01	0.23 ± 0.01	0.21 ± 0.01	0.20 ± 0.03	0.18 ± 0.02	0.17 ± 0.01	0.15 ± 0.01	0.14 ± 0.01	0.12 ± 0.02
*p*-coumaric acid	0.11 ± 0.01	0.09 ± 0.01	0.08 ± 0.01	0.08 ± 0.02	0.06 ± 0.01	0.05 ± 0.01	0.04 ± 0.01	0.03 ± 0.01	0.02 ± 0.01	0.01 ± 0.01
hydroxytyrosol acetate	1.41 ± 0.23	0.60 ± 0.14	0.26 ± 0.09	0.11 ± 0.13	0.08 ± 0.06	0.07 ± 0.01	0.07 ± 0.02	0.07 ± 0.02	0.05 ± 0.01	0.04 ± 0.01
3,4-DHPEA-EDA	233.13 ± 13.99	187.18 ± 14.02	167.37 ± 13.92	150.48 ± 15.92	126.32 ± 13.93	107.39 ± 14.73	107.13 ± 14.69	93.76 ± 14.02	60.17 ± 14.07	41.47 ± 14.73
*p*-HPEA-EDA	117.71 ± 14.02	118.98 ± 6.61	110.09 ± 6.32	97.69 ± 1.34	86.08 ± 6.90	69.93 ± 14.03	61.81 ± 6.98	58.23 ± 4.32	41.10 ± 5.09	30.07 ± 5.40
pinoresinol	5.55 ± 0.14	3.34 ± 0.07	3.16 ± 0.15	3.61 ± 0.05	3.84 ± 0.09	4.28 ± 0.04	4.60 ± 0.20	4.42 ± 0.15	4.03 ± 0.22	4.04 ± 0.13
cinnamic acid	1.95 ± 0.05	1.85 ± 0.01	1.66 ± 0.01	1.60 ± 0.07	1.51 ± 0.14	1.48 ± 0.21	1.40 ± 0.20	1.28 ± 0.01	1.23 ± 0.08	1.14 ± 0.07
acetoxypinoresinol	1.50 ± 0.05	1.50 ± 0.08	1.51 ± 0.07	1.54 ± 0.02	1.46 ± 0.01	1.34 ± 0.04	1.38 ± 0.07	1.44 ± 0.02	1.44 ± 0.01	1.42 ± 0.08
3,4-DHPEA-EA	4.91 ± 1.53	35.44 ± 2.03	32.09 ± 1.21	29.36 ± 1.57	23.60 ± 2.32	21.92 ± 2.03	19.84 ± 0.75	17.46 ± 0.56	12.23 ± 0.62	8.82 ± 1.56
*p*-HPEA-EA	34.21 ± 0.74	18.72 ± 1.32	19.42 ± 0.46	18.99 ± 1.15	18.05 ± 0.71	17.64 ± 0.73	16.97 ± 0.50	16.39 ± 0.48	15.87 ± 0.63	15.09 ± 0.76
luteolin	7.97 ± 0.73	7.67 ± 1.31	6.55 ± 1.46	5.59 ± 1.47	4.83 ± 0.67	4.15 ± 0.81	2.98 ± 0.64	2.07 ± 0.68	1.32 ± 0.65	0.42 ± 0.62
apigenin	2.08 ± 0.06	2.01 ± 0.06	1.92 ± 0.07	1.43 ± 0.04	1.49 ± 0.03	1.46 ± 0.01	0.82 ± 0.04	0.80 ± 0.07	0.64 ± 0.03	0.45 ± 0.02
total concentration	451.25 ± 28.07	381.09 ± 21.39	348.81 ± 20.98	315.62 ± 18.11	273.11 ± 19.87	235.88 ± 28.17	223.78 ± 21.56	202.90 ± 18.53	155.95 ± 12.82	121.38 ± 12.61

a Total concentrations of phenols are also showed.

The fresh samples were stored under the described conditions (see Section 2.1), and they underwent a continuous deterioration of their quality, as it was reflected in the quality parameters (PV, FFA, K270, and K232 and median of the fruity attribute and median of defect), whose time-trend during the storage was already published in a data set.^37^ This data set also includes the information on α-tocopherol, which evolved rapidly in the first 5–10 months. Although this compound also contributes to VOO stability, the latter is largely attributed to phenols.^13,14^ Regarding the concentration of phenols, as reported in Table 1, the total content decreased in the four VOOs along the storage experiment. Table 2 shows the concentration loss expressed as percentage compared to the initial concentration; it was approximately 50%, in the range of 45.76–73.10%. The oil with the highest reduction was VOO4. The maximum concentration at the end of the experiment was found in VOO3 (237.61 mg/kg), whereas VOO1 showed the minimum concentration, 93.47 mg/kg (Table 1). An ANOVA was carried out between three different moments in storage (periods): period 1, at the first stage (0–3 months of storage); period 2, after a year (12–15 months of storage); and period 3, after 2 years (24–27 months of storage). This study confirmed significant differences (*p* < 0.05) of the total concentration of phenols between the three periods mentioned above. Figure S3 shows the plot of the means obtained through the ANOVA study. The regression coefficient (*R*^2^) associated with the concentration change over time (number of storage months) was equal to or higher than 0.95 for the four oils. Table 2 shows the *R*^2^ values as well as the concentration loss. When the concentration values were normalized by the initial concentration (the concentration values of each storage month divided by the initial concentration), the slope of the regression equation (Table 2) was similar for the four oils, −0.0231 ± 0.0027. It would mean around 2% concentration loss every month.

**Table 2:** Regression Equations Explaining the Changes of the Total Concentration of Phenols and the Concentration of Phenols That Computed to the Health Claim over Time (Number of Months, *x*; Concentration of Total Phenols or Concentration of Phenols That Computed to the Health Claim, *y*), Regression Coefficient (*R*^2^), and the Concentration Loss after 27 Storage Months Expressed as Milligrams per Kilogram and as Percentage Compared to the Initial Concentration

parameter	samples	linear regression (0–27 month)			concentration loss	
		regression equation (*y* = *ax* + *b*)	normalized regression^a^ equation (*y* = *ax* + *b*)	*R*^2^	mg/kg	percentage (%)
total phenol	VOO1	*y* = −5.7503*x* + 230.16	*y* = −0.0255*x* + 1.0209	0.95	134.94	59.86
	VOO2	*y* = −6.5283*x* + 340.11	*y* = −0.0193*x* + 1.0036	0.97	155.09	45.76
	VOO3	*y* = −12.171*x* + 531.73	*y* = −0.0228*x* + 0.9942	0.96	297.21	55.57
	VOO4	*y* = −11.287*x* + 423.35	*y* = −0.025*x* + 0.9382	0.98	329.87	73.10
phenols under health claim	VOO1	*y* = −4.787*x* + 198.08	*y* = −0.0250*x* + 1.0357	0.95	110.11	57.57
	VOO2	*y* = −6.4163*x* + 321.99	*y* = −0.0200*x* + 1.0047	0.97	152.26	47.51
	VOO3	*y* = −12.107*x* + 518.79	*y* = −0.0233*x* + 0.9978	0.96	295.43	56.82
	VOO4	*y* = −11.133*x* + 406.72	*y* = −0.0258*x* + 0.942	0.98	328.37	76.05

a Regression equation carried out on the normalized data, in which the concentration values of each storage month are divided by the initial concentration.

The maintenance of a suitable concentration of determined phenolic compounds during the VOO shelf life is of great interest to its nutritional and healthy properties, considering the health claim established by the European Commission. According to the commission regulation (EU) no. 432/2012,^8^ a sample of olive oil can use the health claim if it contains at least 5 mg of hydroxytyrosol and its derivatives (hydroxytyrosol, tyrosol, hydroxytyrosol acetate, tyrosol acetate, 3,4-DHPEA-EDA, *p*-HPEA-EDA, 3,4-DHPEA-EA, and *p*-HPEA-EA)^45^ per 20 g of olive oil, i.e., 250 mg/kg.^8^ At the beginning of storage, all of the oils complied with the limit established by the EU regulation, except VOO1 (Table 1). Thus, VOO1–VOO4 contained 191.25, 320.50, 519.93, and 431.76 mg/kg, respectively. The concentration of phenols that computed to the health claim were higher than the limit of 250 mg/kg until month 10 for VOO2 and month 12 for VOO4, showing that these oils maintained the health claim valid until approximately 1 year, which is usually the maximum VOO shelf life indicated by producers.^46,47^ VOO3, of Picual cultivar, was the oil that complied with the health claim requirements for the longest time (Table 1), reducing its concentration below 250 mg/kg in month 20. The changes over time also followed a linear trend similar to that of the total phenol content (Table 2). Thus, *R*^2^ was also equal or higher than 0.95 and the slope when the data were normalized was also similar, 0.0235 ± 0.0026.

In addition to the sum of different groups of phenols, the changes in the different individual phenols were also studied. The complex phenolic compounds in VOO (secoiridoids), the dialdehydic form of elenolic acid linked to hydroxytyrosol (3,4-DHPEA-EDA), dialdehydic form of decarboxymethyl elenolic acid linked to *p*-HPEA (*p*-HPEA-EDA), and the aldehydic form of elenolic acid linked to hydroxytyrosol (3,4-DHPEA-EA), have been pointed out as powerful antioxidants by many authors,^11,12^ which is related to the stability parameter of VOOs. As reported in Table 1, the time trend of the concentrations of the hydroxytyrosol (3,4-DHPEA-EDA and 3,4-DHPEA-EA) and tyrosol (*p*-HPEA-EDA and *p*-HPEA-EA) derivatives during the storage showed a reduction of their concentration. Table 3 shows the concentration variations of these compounds together with the simple phenols hydroxytyrosol and tyrosol during the storage experiments as well as the regression equations of these concentration changes with respect to storage time. The secoiridoids underwent a mean concentration loss of 56.18%, with a range of 14.41–84.36%. The variation of these compounds followed a linear behavior with *R*^2^ equal or higher than 0.95 in most of the cases for 3,4-DHPEA-EDA, *p*-HPEA-EDA, and 3,4-DHPEA-EA, while *p*-HPEA-EA clearly showed a nonlinear behavior except for VOO3 (Table 3). For the three first compounds, the mean slope of the corresponding regression equations carried out on the normalized data was of −0.0263 ± 0.0050, similar to those found for total phenols (Table 2).

**Table 3:** Regression Equations Explaining the Concentration Changes of the Simple Phenols Hydroxytyrosol and Tyrosol and Their Derivatives (3,4-DHPEA-EDA, 3,4-DHPEA-EA, *p*-HPEA-EDA, and *p*-HPEA-EA) over Time (Number of Months, *x*; Concentration of Each Individual Phenols, *y*), Regression Coefficient (*R*^2^), and the Concentration Gain or Loss after 27 Storage Months Expressed as Milligrams per Kilogram and as Percentage Compared to the Initial Concentration

parameter	samples	linear regression (0–27 month)			concentration gain^b^/loss^c^	
		regression equation (*y* = a*x* + *b*)	normalized regression^a^ equation (*y* = a*x* + *b*)	*R*^2^	mg/kg	percentage
hydroxytyrosol	VOO1	*y* = 0.0675*x* + 0,6619	*y* = 0.1384*x* + 1,3569	0.96	1.78^b^	363.27^b^
	VOO2	*y* = 0.103*x* + 12.949	*y* = 0.008*x* + 0.9998	0.99	2.76^b^	21.31^b^
	VOO3	*y* = 0.1874*x* + 2.6365	*y* = 0.0826*x* + 1.1621	0.98	5.16^b^	227.31^b^
	VOO4	*y* = 0.1651*x* + 1.4237	*y* = 0.1432*x* + 1.2344	0.99	4.74^b^	412.17^b^
tyrosol	VOO1	*y* = 0.087*x* + 2.4184	*y* = 0.0396*x* + 1.0993	0.94	2.35^b^	106.82^b^
	VOO2	*y* = 0.0484*x* + 4.0782	*y* = 0.0127*x* + 1.065	0.89	1.37^b^	35.77^b^
	VOO3	*y* = 0.1642*x* + 3.62	*y* = 0.0474*x* + 1.0445	0.99	4.48^b^	129.11^b^
	VOO4	*y* = 0.0353*x* + 1.4403	*y* = 0.0286*x* + 1.1673	0.90	1.12^b^	91.06^b^
3,4-DHPEA-EDA	VOO1	*y* = −0.9955*x* + 27.795	*y* = −0.0355*x* + 0.9923	0.95	23.63^c^	84.36^c^
	VOO2	*y* = −1.1914*x* + 89.264	*y* = −0.014*x* + 1.0489	0.86	15.15^c^	17.80^c^
	VOO3	*y* = −2.0324*x* + 81.28	*y* = −0.025*x* + 1.0015	0.96	47.44^c^	58.45^c^
	VOO4	*y* = −6.3253*x* + 212.83	*y* = −0.0271*x* + 0.9129	0.97	191.66^c^	82.21^c^
*p*-HPEA-EDA	VOO1	*y* = −2.2232*x* + 79.904	*y* = −0.0296*x* + 1.0644	0.93	48.11^c^	64.09^c^
	VOO2	*y* = −3.3629*x* + 122.64	*y* = −0.0287*x* + 1.0472	0.97	78.09^c^	66.68^c^
	VOO3	*y* = −3.318*x* + 135.92	*y* = −0.0236*x* + 0.9685	0.94	85.07^c^	60.62^c^
	VOO4	*y* = −3.4687*x* + 125.99	*y* = −0.0295*x* + 1.0704	0.98	87.64^c^	74.45^c^
3,4-DHPEA-EA	VOO1	*y* = −1.4029*x* + 53.747	*y* = −0.0262*x* + 1.0047	0.96	33.79^c^	63.17^c^
	VOO2	*y* = −1.8866*x* + 72.695	*y* = −0.0241*x* + 0.9273	0.96	51.74^c^	65.99^c^
	VOO3	*y* = −6.5846*x* + 258.66	*y* = −0.0256*x* + 1.0053	0.97	161.78^c^	62.88^c^
	VOO4	*y* = −1.1569*x* + 39.986	*y* = −0.027*x* + 0.9319	0.98	34.09^c^	79.45^c^
*p*-HPEA-EA	VOO1	*y* = −0.1092*x* + 24.6	*y* = −0.0048*x* + 1.0908	0.28	3.25^c^	14.41^c^
	VOO2	*y* = −0.1245*x* + 20.342	*y* = −0.0055*x* + 0.8938	0.39	5.17^c^	22.72^c^
	VOO3	*y* = −0.3915*x* + 32.683	*y* = −0.0122*x* + 1.0212	0.90	8.23^c^	25.71^c^
	VOO4	*y* = −0.4317*x* + 24.962	*y* = −0.0126*x* + 0.7296	0.51	19.12^c^	55.89^c^

a Regression equation carried out on the normalized data, in which the concentration values of each storage month are divided by the initial concentration.

b Concentration gain during the storage time.

c Concentration loss during the storage time.

Contrary to secoiridoids, the concentration changes of hydrotyrosol and tyrosol showed the reverse situation and they increased their concentration due to the degradation of their complex derivatives.^48,49^ In this case, the mean concentration gains for these two compounds expressed as percentage with respect to the initial concentration was 173.35% and the range was 21.31–412.17%. In fact, the correlation coefficients between hydroxytyrosol and its derivatives (3,4-DHPEA-EDA and 3,4-DHPEA-EA) and tyrosol and its derivatives (*p*-HPEA-EDA and *p*-HPEA-EA) were equal to or higher than −0.95 in all of the cases. In both compounds, the maximum concentration values were found at the end of storage (Table 1). Thus, the concentration of hydroxytyrosol increased at least threefold at the end of the storage, except to VOO2, whose concentration was multiplied by 1.2 (Table 1). Thus, the final concentration ranged from 2.27 to 15.71 mg/kg. On the other hand, the concentration of tyrosol at the end of the storage was 1.36–2.29 times higher than the initial concentration, and the final concentration ranged from 2.35 to 7.95 mg/kg. These results denoted a slower degradation of their complex forms compared with those from hydroxytyrosol. The regression coefficient explaining these changes over time also showed certain linearity with *R*^2^ equal to or higher than 0.95 for hydroxytyrosol and 0.90 for tyrosol (Table 3). The slopes when the regression was carried out with the normalized data were 0.0931 ± 0.0630 and 0.0321 ± 0.0150 for hydroxytyrosol and tyrosol, respectively (Table 3). The standard variation of these slopes was higher than those showed for secoiridoids and total phenols, pointing out a higher variance between samples for these two phenols. On the other hand, these slopes also denoted a slower change of tyrosol compared with that of hydroxytyrosol. In accordance with these results and as other authors have previously reported,^11,50,51^ tyrosol derivatives are maintained for longer time in VOOs than the hydroxytyrosol ones. Therefore, these results seem to point out the higher implication of hydroxytyrosol derivatives in stability compared with tyrosol derivatives.^50,51^

**Figure 2:**
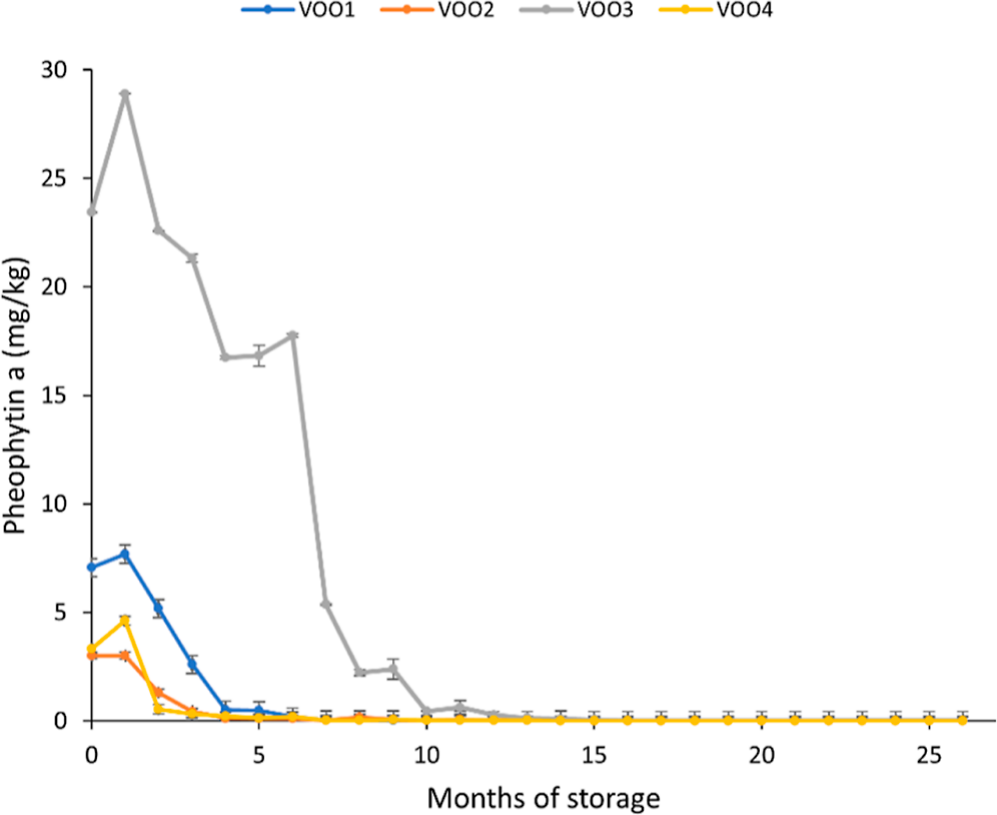
Time-trend of pheophytin *a* concentration during 27 months of storage in the four VOOs. The concentration at time 0, before starting the storage (fresh oils), is also showed. Error bars expressing standard deviation have been included.

With respect to the pigments derived from chlorophyll, pheophytin *a* was analyzed; although other pigments could also have an effect in photooxidation, the main chlorophyll pigment detected in the samples was this compound. Figure 2 shows that the highest concentration of pheophytin *a* in the fresh samples (storage month 0) was found in VOO3 with a value of 23.43 mg/kg, whereas the rest of the oils showed values of 7.06 mg/kg (VOO1), 3.02 mg/kg (VOO2), and 3.31 mg/kg (VOO4). Regarding the time trend of pheophytin *a* content (Figure 2) during the storage experiment under moderate light and temperature condition, the concentration decreased in contrast to other studies carried out at moderate temperature and dark.^18,52^ The pheophytin *a* concentration reached values lower than 1 mg/kg before 13 months of storage. In particular, VOO2 and VOO4 reached this concentration value in the 3rd month of the storage, VOO1 in the 5th month, and VOO3 in the 10th month.

### 3.2 Role of Minor Compounds Activity in Fresh and Stored VOOs Subjected to a Controlled Stress by a Spectroscopic Marker (Mesh Cell-FTIR)

**Figure 3:**
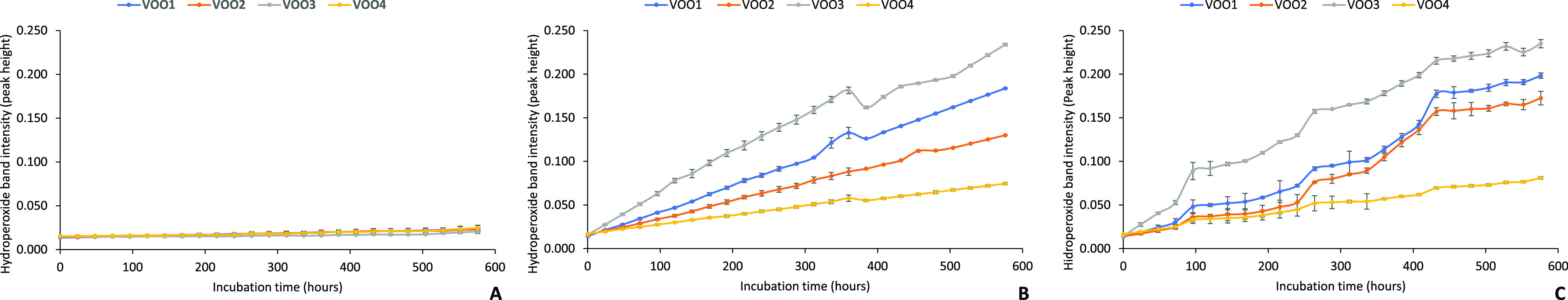
Time trends of the peak height (absorbance units or A.U.) of the spectral band at 3430 cm^–1^ assigned to hydroperoxides when fresh VOOs were subjected to a controlled stress for 576 h of incubation in a mesh cell under three moderate conditions: dark and 35 °C (A), 400 lx and 23 °C (B), and 400 and 35 °C (C). Error bars expressing standard deviation have been included.

A strategy based on mesh cell-FTIR was used to assess the chemical response of fresh VOOs to oxidation when they were subjected to incubation (controlled stress) under moderate conditions, taking into account their minor chemical composition. This accessory allowed to accelerate the oxidation process through VOO incubation^27^ using moderate conditions: dark and 23 °C, 400 lx and 23 °C, and 400 lx and 35 °C. Figure 3 shows the time trend of the intensity (peak height value) of the hydroperoxide band (3430 cm^–1^) when the fresh VOOs were subjected to controlled stress during 576 h of incubation in the mesh cell under the three moderate conditions. The results show that the incubations involving light, with or without heating (35 or 23 °C), were similar between them and promoted the formation of hydroperoxides at much greater extent compared with experiment in the dark and 35 °C (Figure 3). The incubation of the oils on mesh cells can be regarded as a controlled stress in which minor compounds as phenols and pigments have a significant role. The absorbance of the hydroperoxide band at the end of the incubation minus the absorbance before the incubation (henceforth absorbance gain) showed differences between samples that were related with the composition of phenols and pigments. Thus, Figure S4 (Supporting Information) shows the absorbance gain for the four samples incubated under the three conditions. The values of absorbance gain for the hydroperoxide band of the four samples when they were incubated at 400 lx and 23 °C (0.185, 0.159, 0.221, and 0.064 absorbance units or A.U. for VOO1–VOO4, respectively) and 400 lx and 35 °C (0.170, 0.115, 0.218, and 0.058 A.U. for VOO1–VOO4, respectively) showed a clear effect of the pheophytin *a* content in VOO3. Thus, the latter was the oil with the highest initial concentration of pheophytin *a* (Figure 2) and also showed the highest values of absorbance gain of the hydroperoxide band under both conditions (Figure S4—Supporting Information). However, this difference with respect to the rest of samples was not observed when they were incubated in the dark at 23 °C (0.010, 0.008, 0.006, and 0.010 A.U.). In this case, the effect of pigments, which can act as antioxidants in the dark,^20^ was observed by the slope of the time trend of the hydroperoxide band in VOO3 (0.064 A.U.), which was lower than the rest of the oils (0.185, 0.159, and 0.221 A.U. for VOO1, VOO2, and VOO4 respectively) (Figure 3). Furthermore, the order of total phenols for VOO1, VOO2, and VOO4 (Table 1) also seemed to influence the order of the absorbance gain observed in these samples when they were incubated at 400 lx and 23 °C and 400 lx and 35 °C (Figure S4, Supporting Information).

A further study with mesh cell incubation was carried out using the oil samples collected during 27 months of storage and repeating the mesh cell incubation in each one of them. This study was focused on assessing the antioxidant or pro-oxidant activity of the minor compounds present in VOO in connection with the changes of the concentration of these compounds during the shelf life. During VOO shelf life, minor changes in the chemical composition of the oils may lead to variations in stability^35^ and, therefore, a different response of the aged oils to storage conditions compared to fresh oils can be expected.

**Figure 4:**
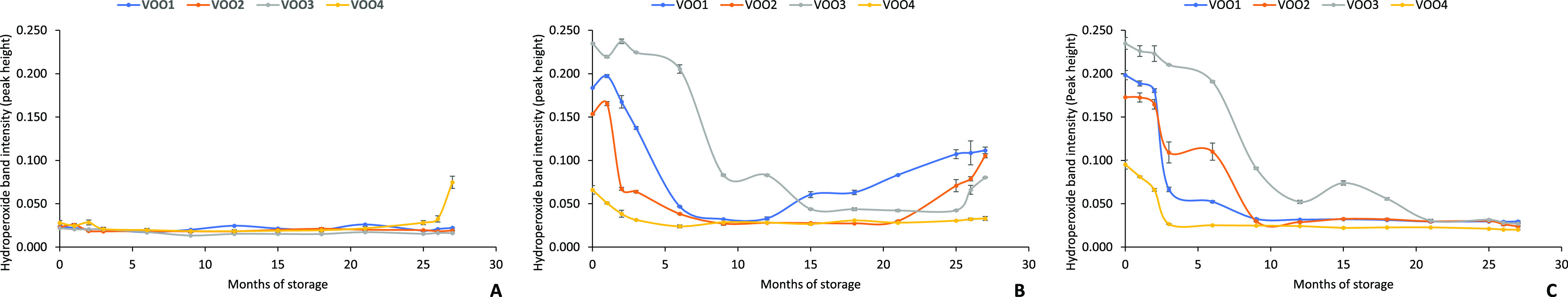
Intensity of the hydroperoxide band (expressed as peak height value, absorbance units) reached by the storage samples after 576 h of mesh cell incubation under the three different conditions: dark and 35 °C (A), 400 lx and 23 °C (B), and 400 lx and 35 °C (C). Error bars expressing standard deviation have been included.

Figure 4 shows the intensity reached by the hydroperoxide band at the end of the 576 h of incubation carried out with the samples collected during the storage. Like it was observed through the incubation of the fresh samples, Figure 4 shows that the experiments carried out with light (Figure 4B,C) always showed more variability in the hydroperoxide band intensity compared with the spectral signals obtained after the incubation in the dark and 35 °C. Furthermore, in these two cases, the changes in the intensity are observed from the first months, as it was observed in both phenol and pheophytin *a* concentrations. However, these changes in concentration during storage seemed to be not enough for producing changes in the absorption of the hydroperoxide band when the oils were incubated in the dark at 35 °C. When light was applied at two different temperatures (35 and 23 °C), the hydroperoxide band showed the maximum absorbance values in the first months of storage (Figure 4B,C), and this value was reduced until the 6th storage month in VOO1, VOO2, and VOO4 and 12th storage month in VOO3. These two moments approximately matched with the moments when the pheophytin *a* concentration reached the minimum concentration values in these oils (Figure 2). These results revealed that the prooxidant effect of pigments was able to mask the protective effect of the phenolic compounds in the presence of diffuse light. After this stage, the time trend of the hydroperoxide band intensity was getting constant during the following months, which is attributed to the antioxidant activity of phenols after the cessation of the prooxidant action of pheophytin *a* (<1 mg/kg).

The main differences between the two experiments carried out with light (400 lx and 23 °C and 400 lx and 35 °C) were the response of the hydroperoxide band during the last months of storage (Figure 4B,C). After 1 year of storage, the incubation under 400 lx and 23 °C again induced hydroperoxide formation at different moments depending on the oil (Figure 4B). VOO1 showed an increase of the hydroperoxide band intensity in 12th and subsequent months, followed by VOO2 in the 21st month. The oils VOO3 and VOO4 were the last oils showing variation in the intensity of this band after the 25th month. This order of response of the hydroperoxide band was the same as the order of the initial concentration of total phenol content (from low to high): VOO1 < VOO2 < VOO4 < VOO3 (Table 1). According to these results, the protective effect of phenols was in accordance with their concentration, taking into account that the complex phenols were the most abundant in the samples. Analyzing the total phenol concentrations in these months (Table 1), it could be inferred that a concentration loss of 35% (e.g., in VOO1 in the 12th month) or more may produce an increase of the hydroperoxide band (Table 1). The increment of the hydroperoxide band was less evident in VOO4, which showed only a slight increment of the hydroperoxide band from month 25. The increment of the hydroperoxide band in the last storage months was not observed when the oils were incubated at 400 lx and 35 °C, probably due to the effect of temperature on the decomposition rate of hydroperoxides.^53^

**Figure 5:**
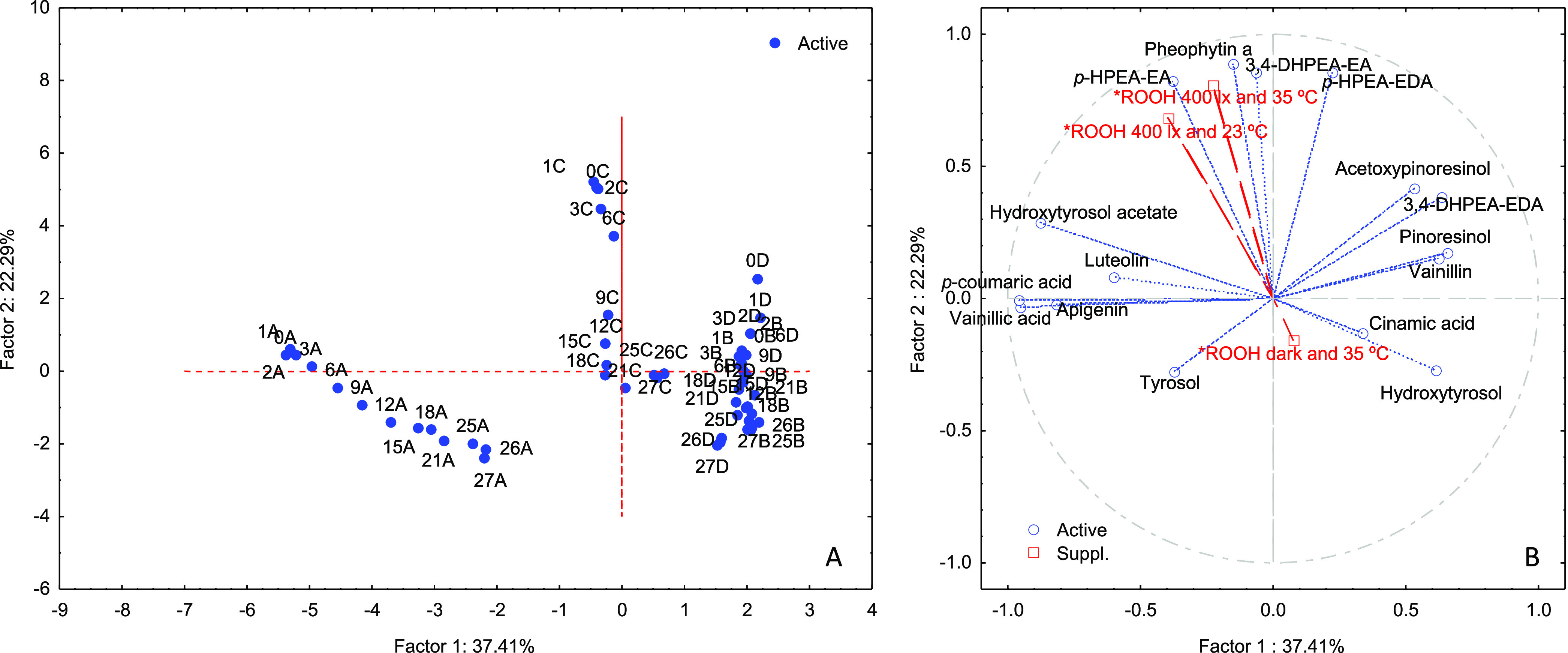
Scores (A) and loadings (B) plots obtained through the PCA performed with the concentration values of phenolic compounds and pheophytin *a* quantified in the stored samples during the storage. The hydroperoxide band intensity of the stored samples after 576 h of controlled stress in the mesh cell was included in the PCA as supplementary variables. Note: the letters in the score plot indicate the different samples (A, VOO1; B, VOO2; C, VOO3; D, VOO4) and the number indicates the storage months (0–27).

Given the complexity of the relationship between the phenols, pheophytin *a*, and the deterioration of the oil under moderate conditions, a PCA was performed with the concentration of these compounds quantified in the stored samples during storage. Furthermore, the hydroperoxide band intensity of the stored samples after 576 h of controlled stress in the mesh cell was included in the PCA just as supplementary variables in order to study their projection on the chromatographic results. Figure 5 shows the scores and loadings plots obtained through the PCA. The score plot (Figure 5A) showed the grouping of the samples according to their cultivar along PC1, whereas PC2 explained the distribution of the samples in accordance with their age. On the other hand, the analysis of the projection of the hydroperoxide band intensity in the loadings plot (Figure 5B) revealed that under controlled stress with light conditions, the antioxidant activity seems to be related to the complex secoiridoid phenols derived from oleouropein, whereas the incubation carried out in the dark with moderate heating showed to be more related with the activity of simple phenols (hydroxytyrosol and tyrosol) and some acid phenols (vanillic and cinnamic acids). Furthermore, the pheophytin *a* showed to be clearly involved in the oxidation changes in the collected samples subjected to controlled stress under light (400 lx and 23 °C and 400 lx and 35 °C), but it did not show relation with the incubation performance in the dark at 35 °C.

The results explained above evidenced the relations between secoiridoid derivatives and the hydroperoxide FTIR band when light conditions were applied (Figure 5). In order to study further this relationship, a PLS model was developed with the mesh cell-FTIR spectra (3587–3047 cm^–1^ region assigned to the hydroperoxide band) and the concentration of phenols. Although phenols do not produce distinctive signals in the FTIR spectra, these models were built to examine with a multivariate perspective if the changes measured in the spectra evolved with the same time trend as phenols during the VOO storage. The phenols considered in this model were those covered by the health claim established by the EU (hydroxytyrosol, tyrosol, hydroxytyrosol acetate, tyrosol acetate, 3,4-DHPEA-EDA, *p*-HPEA-EDA, 3,4-DHPEA-EA, and *p*-HPEA-EA). A model was built per VOO sample in order to consider different cultivars and oils with different concentrations of antioxidants and prooxidants. The calibration models were performed using the spectra obtained after 576 h of incubation in the mesh cell under the two experiments carried out with light (400 lx and 23 °C and 400 lx and 35 °C), which were those providing the most significant changes.

The results of the PLS models are listed in Table 4. The model performance for the concentration of the compounds computed for the health claim showed the best results when the VOOs were subjected to the controlled stress under 400 lx and 23 °C. In this experiment, the four stored oils showed *R*_CV_^2^ > 0.87 and *R*_P_^2^ > 0.84. Furthermore, they showed RPD_p_ values higher than 2.50. These results prove the relationship between both kinds of information, FTIR spectra evolving due to the oil oxidation and the changes in phenol concentrations. In contrast, the PLS model obtained from the controlled stress under 400 lx and 35 °C showed low accuracy and poor fit of the spectral data in VOO1 and VOO2 due to its low values of *R*_P_^2^ and RPD_P_ (Table 4). This sample was the fresh VOOs with the lowest concentration of total phenols, which may have some impact on the model. Nevertheless, this model showed better resolution for samples with higher initial concentration of total phenols, as VOO3 and VOO4, which showed good results (*R*_P_^2^ > 0.90 and RPD_P_ > 2.90) (Table 4).

**Table 4:** Characteristics of the PLS Models for the Representation of the Changes Underwent in the Phenolic Compounds that Compute for the Health Claim Established by the European Union during VOO Storage under Moderate Conditions^a^

model	incubation condition	sample	LVs	calibration			prediction		
				*R*_CV_^2^	RMSECV	RPD_cv_	*R*_P_^2^	RMSEP	RPD_P_
health claim phenolic compounds	400 lx and 23 °C	VOO1	5	0.87	17.05	2.87	0.85	13.99	2.84
		VOO2	6	0.95	13.77	4.66	0.87	19.75	2.67
		VOO3	2	0.90	38.45	3.26	0.84	48.23	2.69
		VOO4	4	0.89	34.69	3.15	0.89	27.52	3.14
	400 lx and 35 °C	VOO1	3	0.69	26.43	1.59	0.62	24.39	1.72
		VOO2	7	0.88	21.61	2.26	0.72	28.36	1.92
		VOO3	4	0.96	23.77	5.27	0.96	49.49	4.62
		VOO4	7	0.98	12.96	8.24	0.90	28.76	2.93

a PLS models were built using the spectra obtained during the VOO storage and after 576 h of mesh cell incubation under 400 lx and 23 °C and 400 lx and 35 °C.

The effect of the storage under controlled temperature (23 ± 7 °C) and light (1000 lx) conditions on the chemical composition of the VOOs was identified from the beginning of the experiment, generating significant changes in the quality parameters, phenolic composition, and the pheophytin *a* concentration after 27 months of storage. Despite these changes, the VOOs kept the levels of the phenol content described in the European health claim during at least 1 year of storage under conditions not far from those found in a supermarket shelf.

The controlled stress of the fresh and stored VOOs by incubation under different moderate conditions revealed the different roles of phenols and pheophytin *a* in the formation of hydroperoxides depended on the incubation conditions applied. The mesh cell incubations carried out with light revealed that the pro-oxidant effect of pheophytin *a* was able to mask the protective effect of phenolic compounds during the first months of storage and the antioxidant effect of phenols was shown to be more relevant when the pheophytin *a* concentration was lower than 1 mg/kg. The differences in the stability of the studied samples suggested that it is not possible to establish a low phenol concentration threshold, at which the protective effect ceases in all cases. However, it was observed in the studied samples that the protective effect of phenols diminished when the residual concentration was less than 60% of the initial concentration. On the other hand, the mesh cell incubation under mild heating in the dark practically showed no response of the hydroperoxide band intensity, which may be due to the synergic effect of the antioxidant activity of phenol compounds and pheophytin *a*.

The PLS models showed basic agreement in the time trends of the spectral changes and the variations of the phenolic content. These results illustrate the importance of considering the balance between antioxidants/prooxidants in conjunction with the storage conditions (e.g., dark or transparent bottles) to verify the maintenance of healthy and sensory properties prior to the consumption. On the other hand, this study also proves the potential application of FTIR spectroscopy in the assessment of the oxidation state of VOO samples in the industry with enough time resolution to allow the tracking of chemical changes over time. This technique, with the mesh cell approach, would also allow a better understanding of the role of antioxidants and prooxidants in the quality evolution of the samples for a proper management of VOO in the supply chain.

## Supporting Information Available

Chromatograms obtained in the phenolic compound analysis; codes indicating the phenolic compounds identified: codes: 1, hydroxytyrosol; 2, tyrosol, 3, *p*-hydroxyphenylacetic acid (internal standard), 4, vanillic acid; 5, vanillin; 6, *p*-coumaric acid; 7, hydroxytyrosol acetate; 8, *o*-coumaric acid (internal standard); 9, 3,4-DHPEA-EDA; 10, *p*-HPEA-EDA; 11, pinoresinol; 12, cinnamic acid; 13, acetoxypinoresinol; 14, luteolin; 15, 3,4-DHPEA-EA; 16, apigenin; and 17, *p*-HPEA-EA; A, elenolic acid; chromatogram obtained in the pigment analysis for VOO1(A); zoomed-in view of the chromatogram to identify compound 3; codes: 1, pheophytin *a*; 2, pheophytin *a*′; and 3, pyropheophytin *a*; results obtained through ANOVA performed with the total concentration of phenols during the whole storage (27 months) for each VOO; absorbance gain of the hydroperoxide band (3430 cm^–1^) of the fresh VOOs when they were subjected to control stress through 576 h of incubation in a mesh cell under the three moderate conditions (PDF)

## Abbreviations

VOO: virgin olive oil
FTIR: Fourier transform infrared
FFA: free fatty acid
PV: peroxide value
DTGS: deuterated triglycine sulfate
ANOVA: analysis of variance
PCA: principal component analysis
PLS: partial least squares
CV: cross-validation
P: independent test samples
*R*_CV_^2^: regression coefficient for cross-validation
*R*_P_^2^: regression coefficient for independent test samples
RMSECV: root-mean-square error for cross-validation
RMSEP: root-mean-square error for the prediction error
RPD_CV_: relative predictive deviation for cross-validation
RPD_P_: relative predictive deviation for prediction
3,4-DHPEA-EDA: dialdehydic form of elenolic acid linked to hydroxytyrosol
*p*-HPEA-EDA: dialdehydic form of decarboxymethyl elenolic acid linked to *p*-HPEA
3,4-DHPEA-EA: aldehydic form of elenolic acid linked to hydroxytyrosol
*p*-HPEA-EA: aldehydic form of elenolic acid linked to tyrosol
A.U.: absorbance unit
