# Supplementary material for: Gradual Changes of the Protective Effect of Phenols in Virgin Olive Oils Subjected to Storage and Controlled Stress by Mesh Cell Incubation
Source: J Agric Food Chem. 2023 Oct 11;71(42):15732–44. doi: 10.1021/acs.jafc.3c04169 (PMC10603807; doi:10.1021/acs.jafc.3c04169)
Supplement: Supplementary file 1 — jf3c04169_si_001.pdf [file jf3c04169_si_001.pdf]

SUPPORTING INFORMATION

Gradual changes of the protective effect of phenols in virgin olive oils subjected to storage and controlled stress by mesh cell incubation

Ana Lobo-Prieto[†,‡], Noelia Tena[†], Ramón Aparicio-Ruiz[†], María Teresa Morales[†], Diego Luis García-González[ª]*

[†]Departamento de Química Analítica, Facultad de Farmacia, Universidad de Sevilla, Prof. García González, 2, 41012, Sevilla, Spain.

[‡]Pablo de Olavide University, Ctra. de Utrera, km 1, 41013 Sevilla, Spain.

[ª]Instituto de la Grasa (CSIC), Edificio 46, Ctra. de Utrera, km 1, 41013 Sevilla, Spain.

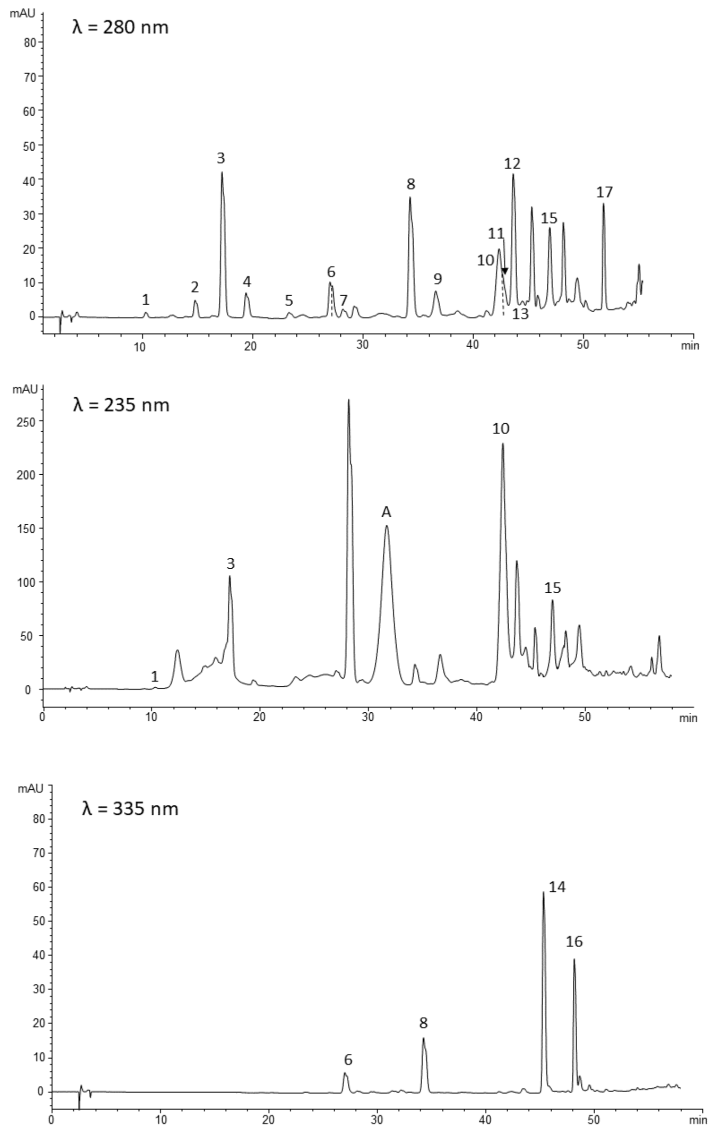

Figure S1. Chromatograms obtained in the phenolic compounds analysis. The codes indicated the phenolic compounds identified. Codes: 1, hydroxytyrosol; 2, tyrosol, 3, *p*-hydroxyphenylacetic acid (internal standard), 4, vanillic acid; 5, vanillin; 6, *p*-coumaric acid; 7, hydroxytyrosol acetate; 8, *o*-coumaric acid (internal standard); 9, 3,4-DHPEA-EDA; 10, *p*-HPEA-EDA; 11, pinoresinol; 12, cinnamic acid; 13, acetoxypinoresinol; 14, luteolin; 15, 3,4-DHPEA-EA; 16, apigenin; 17, *p*-HPEA-EA; A, elenolic acid.

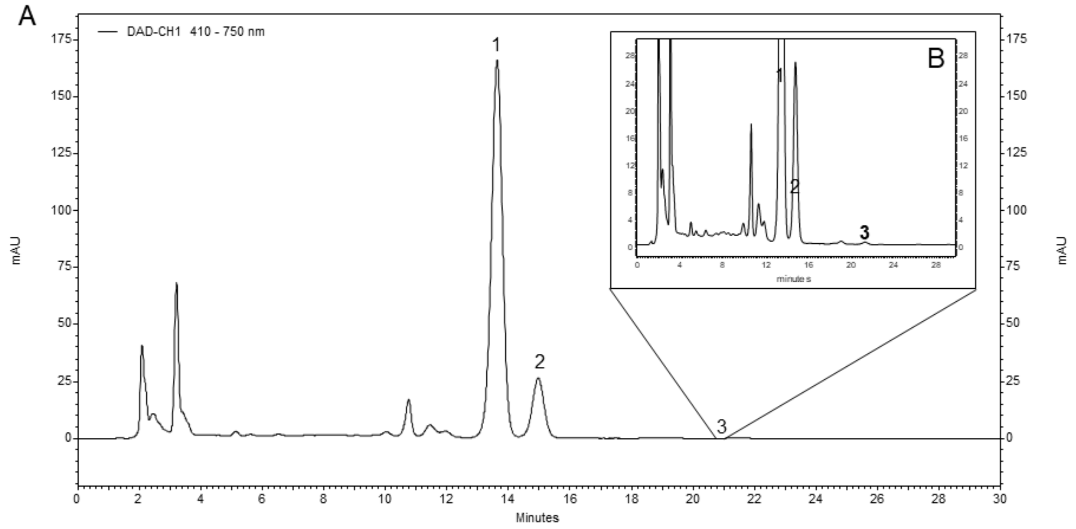

Figure S2. Chromatogram obtained in the pigments analysis for VOO1(A), Zoom of the chromatogram to identify compound 3. Codes: 1, pheophytin a; 2, pheophytin a'; 3, pyropheophytin a.

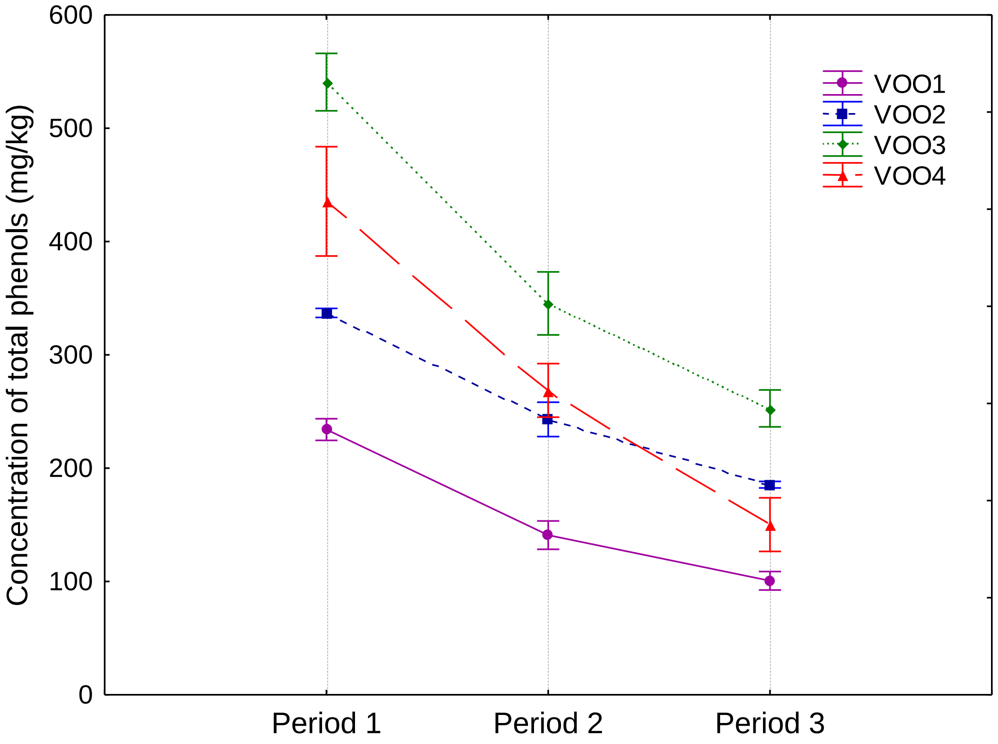

Figure S3. Results obtained through ANOVA performed with the total concentration of phenols during the whole storage for each VOO. Plot of the means of total content of phenols obtained between period 1 (0 – 3 months), period 2 (12 - 15 months) and period 3 (24 – 27 months). The standard deviations obtained from the different periods are also shown.

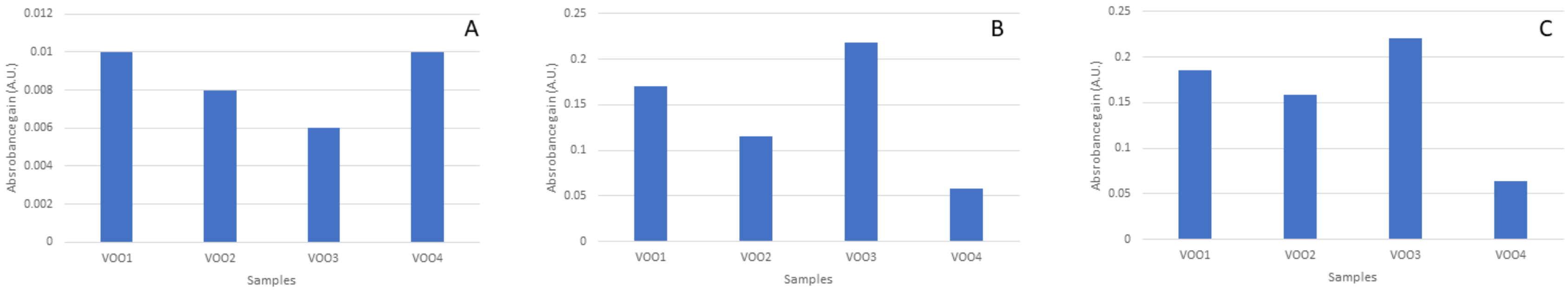

Figure S4. Absorbance gain of the hydroperoxide band (3430 cm^{-1}) computed by subtracting the absorbance values of the hydroperoxide band at the end of the incubation minus the absorbance before the incubation under three different conditions: dark and 35 °C (A), 400 lx and 23 °C (B), and 400 lx and 35 °C (C).
